# Frailty syndrome in older adults: Research advances and management strategies

**DOI:** 10.1016/j.isci.2026.116264

**Published:** 2026-06-09

**Authors:** Yu Guo, Yuxin Hu, Ruoyu Chen, Yiwen Yang, Chunhong Hu, Hui Yao, Yunyan Su

**Affiliations:** 1 Department of Radiology, The First Affiliated Hospital of Soochow University, Suzhou 215006, P.R. China; 2 Department of General Surgery, The First Affiliated Hospital of Soochow University, Suzhou 215006, P.R. China

**Keywords:** health sciences, medicine, medical specialty, gerontology

## Abstract

Frailty represents a critical clinical syndrome characterized by diminished physiological reserve and heightened vulnerability to stressors, serving as a robust predictor of disability and mortality in the aging population. This review comprehensively synthesizes current evidence regarding the biological mechanisms, evolving assessment tools, and evidence-based management strategies for frailty. Complex multisystem dysregulation is identified as the primary driver of this syndrome, with a particular focus on metabolic-immune imbalances and muscle-mitochondrial axis dysfunction. While traditional clinical assessment models remain diagnostic staples, multi-omics profiling and digital biomarkers, including those derived from wearable sensors, are emerging as transformative tools for earlier and more precise risk stratification. Regarding clinical translation, multimodal interventions that integrate resistance exercise, nutritional optimization, and polypharmacy management constitute the cornerstone of current care. However, the future of geriatric medicine necessitates a paradigm shift beyond descriptive diagnosis. Ultimately, mechanism-driven precision interventions and the integration of digital health technologies are proposed to not merely manage frailty but to quantify and enhance systemic resilience for healthy longevity.

## Graphical abstract

**Figure:**
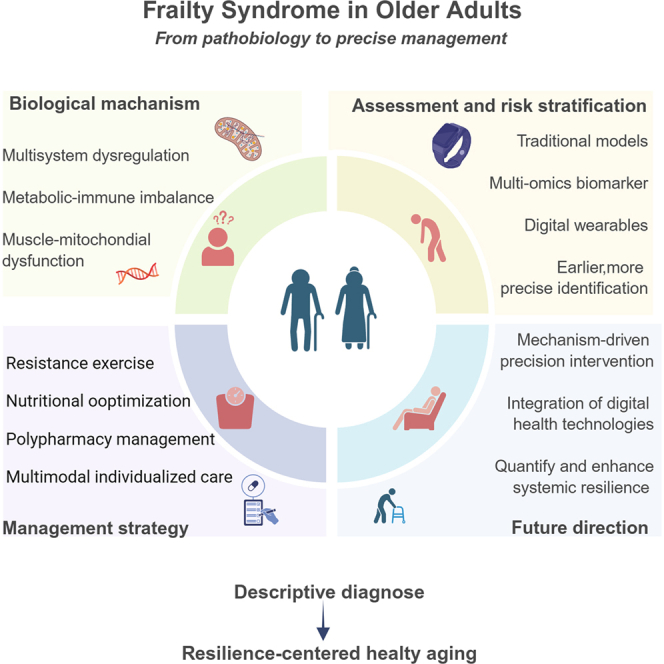

## Introduction

Frailty is a syndrome arising from a marked decline in physiological reserve across multiple organ systems, resulting in increased susceptibility to a range of adverse health outcomes, such as falls, mobility impairment, functional dependence, and disability.^1^ The presence of frailty often amplifies the complexity and severity of coexisting medical conditions in older adults. Consequently, the accurate identification and management of frailty are pivotal for advancing the goals of healthy aging and for constructing sustainable, effective healthcare delivery models for our rapidly aging global population.

Epidemiological data establish the significant burden of frailty, affecting approximately 10–15% of community-dwelling older adults. This prevalence escalates considerably, exceeding 40%, among hospitalized and institutionalized individuals. Furthermore, this syndrome disproportionately impacts women and the oldest-old, underscoring the critical imperative for early detection and precisely tailored interventions.^2^^,^^3^^,^^4^^,^^5^^,^^6^

Owing to its multifactorial etiology, research on frailty has evolved to encompass a wide spectrum of investigative domains, spanning fundamental biological mechanisms, clinical assessment tools, and preventive interventions. Reflecting this breadth, the research paradigm is progressively evolving from initial descriptive phenotyping to a more nuanced, mechanism-driven stratification of the syndrome, with the ultimate goal of enabling dynamic and precisely targeted interventions. Within this conceptual framework, this review systematically synthesizes the extant literature to argue that the strategic integration of multi-omics profiling with digital biomarkers holds transformative potential for the early detection and interception of pre-frailty. More fundamentally, the dynamic, real-world data afforded by these tools enable a paradigm shift that is reshaping geriatric medicine: moving beyond the static diagnosis of frailty toward the quantification of systemic resilience—the capacity to recover from physiological stressors. Throughout this review, we explore how integrating insights from multi-omics, digital biomarkers, and clinical assessment can lay the groundwork for this resilience-centered approach to healthy aging.

## Methodology

### Search strategy and data sources

A targeted literature search was conducted across three electronic databases: PubMed, Embase, and Web of Science, for relevant English-language articles published from inception until March 2026. The search strategy was designed to identify publications encompassing the concepts of frailty, aging populations, biomarkers, assessment, and interventions. The core search syntax used for PubMed is detailed later in discussion, with analogous strategies adapted for Embase and Web of Science:

(frailty [MeSH Terms] OR frail elderly [MeSH Terms] OR “frailty syndrome” OR frail) AND (aging [MeSH Terms] OR “older adults” OR elderly OR “geriatric”) AND (biological markers [MeSH Terms] OR biomarker OR inflammation OR sarcopenia OR cognition) AND (assessment OR screening OR “clinical tools” OR diagnosis) AND (intervention OR management OR prevention OR therapy).

### Inclusion and exclusion criteria

To maintain a high standard of evidence, we applied the following inclusion criteria: (1) peer-reviewed original research, systematic reviews, meta-analyses, and internationally recognized clinical guidelines; (2) studies primarily focusing on older adult populations (especially ≥65 years); and (3) articles published in English. We excluded non-English publications, single-case reports, conference abstracts without full text available, and studies focusing on pediatric cohorts or geriatric syndromes lacking a pathophysiological overlap with frailty.

### Screening process and selection of niche studies

The initial search results underwent a two-stage screening process. First, titles and abstracts were screened for relevance to the overarching themes of frailty diagnosis, mechanisms, and management. Second, full-text articles were appraised. Because a primary aim of this review is to map the paradigm shift from static frailty to dynamic resilience, the final selection of reference list was not based on a rigid algorithmic flowchart (e.g., PRISMA), but rather on thematic relevance, clinical importance, conceptual representativeness, and overall evidentiary value.

Specifically, the selection of “niche” studies was guided by the following principles:

Biomarkers and Omics: We prioritized recent longitudinal studies and systematic reviews that represent key mechanistic pathways, emerging diagnostic approaches, or clinically relevant technological developments.

Imaging and Wearables: We selected studies that are relevant to the conceptual framework of the review and informative for understanding current and future assessment strategies.

Seminal, highly cited publications were retained to establish historical context for traditional models, while priority was given to the most recent literature (published within the last five years) to accurately capture cutting-edge trends in precision interventions.

## Phenotypical classification of frailty

To navigate the evolving literature on frailty, it is essential to distinguish among the various conceptual and operational terms used in the field. Box 1 provides a conceptual roadmap for the terminology utilized throughout this review, ensuring clarity when discussing foundational models, clinical tools, and emerging phenotypes.

Box 1 **Conceptual clarification of frailty-related terms**

• **Theoretical Model**: A foundational conceptual framework explaining the underlying nature of frailty (e.g., the physical phenotype model, the cumulative deficit model).
• **Operational Definition**: The specific criteria or clinical tools used to measure a theoretical model in practice (e.g., the Fried Frailty Phenotype criteria, the frailty index).
• **Risk Factor**: A distinct clinical condition, exposure, or biological variable that increases the likelihood of developing frailty (e.g., metabolic syndrome and chronic inflammation).
• **Subphenotype**: A finer-grained, data-driven classification within the broader frailty syndrome, grouping individuals who share specific biological, clinical, or functional features (e.g., metabolic frailty, active vs. non-active frailty).
• **Multidomain Construct**: An expanded conceptualization of vulnerability that incorporates physical, cognitive, psychological, and/or social domains to capture the full complexity of aging.

### Primary theoretical models

Frailty syndrome is predominantly conceptualized through two primary theoretical paradigms: the physical phenotype model and the cumulative deficit model.^7^^,^^8^ The physical phenotype model characterizes frailty as an independent biological syndrome resulting from diminished physiological reserve, manifesting primarily through physical declines in mobility, strength, and energy. This model identifies a distinct subset of vulnerable older adults and forms the basis for operational tools such as the Fried Frailty Phenotype.^8^

Conversely, the cumulative deficit model views frailty as a state of vulnerability arising from the progressive accumulation of health deficits across multiple physiological systems over time. Rather than a discrete physical syndrome, it conceptualizes frailty as a quantifiable proportion of accumulated symptoms, diseases, and functional impairments, operationalized clinically through tools such as the frailty index (FI).^7^^,^^9^

### Domain-specific extensions

Beyond these foundational models, several domain-specific and multidomain constructs have been proposed to broaden frailty assessment. Cognitive frailty is defined as the co-occurrence of cognitive impairment and physical frailty in the absence of concurrent Alzheimer’s disease or other dementias.^10^ Research on cognitive frailty is increasingly focused on its link to dementia risk, leading to the delineation of two clinical subtypes: potentially reversible cognitive frailty [physical frailty with mild cognitive impairment (MCI)] and reversible cognitive frailty (physical frailty with pre-MCI subjective cognitive decline).^11^^,^^12^ Notably, cognitive frailty may be linked to the progression of physical frailty, potentially mediated by brain aging processes that accelerate physical decline.^13^^,^^14^

Moreover, social frailty has emerged as another multidomain construct, encompassing reduced social participation, weakened support networks, and diminished interpersonal interactions. It is increasingly recognized for its importance in exacerbating both physical decline and cognitive deterioration.^15^^,^^16^

### Data-driven subphenotypes

Recent studies have applied data-driven and machine learning approaches to identify finer-grained subphenotypes within frail populations. For instance, Liu et al.^17^ identified three distinct subphenotypes in an older cohort: non-active (dominated by exhaustion and weakness), active (characterized by slowness), and low physical activity, while the active subgroup exhibited the most unfavorable profiles in survival and cognition. Moreover, Mamchur et al.^18^ employed machine learning on data from older individuals to propose etiologically distinct aging phenotypes, including multimorbid frailty, cognitive frailty, functional frailty, and metabolic frailty. It is important to distinguish the concept of “metabolic frailty” here as a subphenotype from the epidemiological concept of metabolic syndrome (MetS); the former describes a specific cluster of patients grouped by shared metabolic and phenotypic features, while the latter is a risk factor (discussed in Section 4).

The physical frailty phenotype was intentionally designed to be a narrow, focused measure of physical function, with a clear and proven clinical rationale. Conversely, cumulative deficit models such as the FI are inherently multidimensional by definition. Therefore, rather than viewing these traditional models as insufficient, emerging approaches—such as cognitive frailty, social frailty, and data-driven subphenotypes—are best understood as expanding upon these foundational conceptualizations.

These newer models reflect a broader understanding of vulnerability and risk that incorporates additional interacting domains. The emerging approaches, such as cognitive frailty, social frailty, and data-driven subphenotypes based on these foundational conceptualizations, align perfectly with the core philosophy of the Comprehensive Geriatric Assessment (CGA), underscoring that a multidomain, integrated approach is essential to fully screen, understand, and address the complex risks facing older individuals.

## Pathophysiological mechanisms

**Figure 1:**
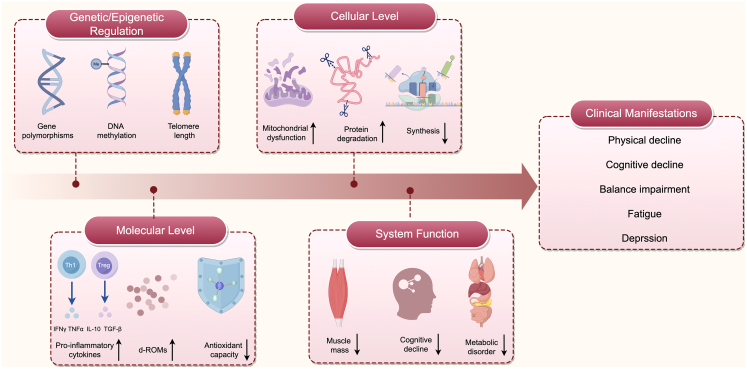
Schematic diagram of the pathophysiological pathways of frailty This schematic illustrates the progression from upstream genetic and epigenetic regulation to molecular, cellular, and system-level dysfunction in frailty. Genetic polymorphisms, DNA methylation, and telomere shortening may interact with inflammatory activation, oxidative stress, reduced antioxidant capacity, mitochondrial dysfunction, increased protein degradation, and impaired protein synthesis. These alterations contribute to declines in muscle mass, cognitive function, and metabolic homeostasis, ultimately leading to clinical manifestations such as physical decline, cognitive decline, balance impairment, fatigue, and depression.

Frailty reflects a state of diminished physiological reserve, resulting in heightened vulnerability to adverse health outcomes upon exposure to stressors. Figure 1 illustrates the conceptual framework of frailty pathophysiology, outlining the progression from genetic and molecular alterations to clinical manifestations. The development of frailty involves complex multisystem interactions, primarily encompassing the following aspects.

### Metabolic-immune imbalance

#### Oxidative stress cascade

Cross-sectional evidence consistently links elevated oxidative stress biomarkers with the frailty phenotype. The core disturbance is a systemic imbalance between pro-oxidant species and antioxidant defenses, culminating in widespread macromolecular damage. Specifically, frail older adults exhibit marked elevations in global oxidative burden, quantified by markers such as derivatives of reactive oxygen metabolites (d-ROMs), lipid peroxidation products (e.g., 8-isoprostanes and malondialdehyde), DNA oxidation adducts (e.g., 8-hydroxy-2′-deoxyguanosine), and protein carbonylation. These biochemical alterations are functionally consequential, correlating with clinical measures such as reduced gait speed.^19^^,^^20^ Within skeletal muscle, excess reactive oxygen species directly stimulate proteolysis, primarily by activating the ubiquitin-proteasome degradation pathway, which drives the loss of muscle mass and contractile function.^21^ Concurrently, key components of the endogenous antioxidant system are depleted in frailty, including plasma total thiols (reflecting redox buffering capacity) and bilirubin, though assessments of total antioxidant capacity have yielded conflicting results.^22^^,^^23^

#### Chronic inflammatory network activation

Chronic low-grade inflammation, commonly referred to as “inflammaging,” is a key mechanism that connects aging with the development of frailty.^24^ This process is largely driven by a widespread dysregulation of cytokine networks. Pro-inflammatory cytokines contribute to frailty through both direct and indirect pathways: directly by accelerating protein breakdown, and indirectly by disrupting anabolic signaling and metabolic balance. Mechanistically, interleukin-1β (IL-1β) can upregulate major atrophy-related genes such as Atrogin-1 and MuRF1 at the transcriptional level, while tumor necrosis factor alpha (TNF-α) enhances glucocorticoid-induced catabolism.^25^ The causal role of inflammatory dysregulation is supported by genetic animal models; for example, mice with chronic nuclear factor kappa B (NF-κB) activation or those deficient in the anti-inflammatory cytokine interleukin-10 (IL-10) develop premature aging phenotypes accompanied by sarcopenia and frailty.^26^

Clinically, cross-sectional evidence consistently demonstrates elevated circulating levels of inflammatory mediators, including interleukin-6 (IL-6), C-reactive protein (CRP), TNF-α, and homocysteine, in frail compared to robust older adults.^27^ The predictive utility of inflammation may be enhanced by using composite biomarker panels (e.g., CRP, P-selectin, and interferon-gamma-inducible protein 10) rather than relying on single markers. Longitudinal data, however, reveal nuances in these associations; while levels of CRP and fibrinogen show more consistent relationships with the progression of the FI over time, the long-term predictive value of IL-6 and interleukin-8 (IL-(8) appears less robust.^22^ Reflecting the complexity of the immune response, the pan-immune inflammation value (PIV)—a composite index derived from neutrophil, monocyte, platelet, and lymphocyte counts—has recently emerged as a promising systemic immune biomarker. Elevated PIV levels (with a median cutoff of 372) have been associated with behavioral patterns known to predispose individuals to frailty.^28^

### Muscle-mitochondrial axis dysfunction

Sarcopenia, the age-related loss of skeletal muscle mass and function, constitutes a central pathophysiological component of physical frailty. This clinical phenotype is associated with distinct circulating biomarker profiles, primarily categorized into an inflammatory signature (e.g., elevated pro-inflammatory fatty acids, IL-6, and IL-6/IL-10 ratio) and a muscle metabolic signature (e.g., altered amino acids and elevated 3-methylhistidine, a marker of myofibrillar breakdown), both of which strongly correlate with frailty.^29^ The underlying pathology involves a triad of deficits: dysregulated muscle protein turnover, loss of muscle mass, and a pro-inflammatory intramuscular milieu, which is further exacerbated by nutritional deficiencies as indicated by low levels of albumin and hemoglobin.^29^^,^^30^^,^^31^

Mitochondrial dysfunction is a critical mechanism within this axis, driving the syndrome forward. Frail individuals exhibit systemic disturbances in cellular energy metabolism, including inefficiencies in glycolysis and the tricarboxylic acid (TCA) cycle, resulting in reduced ATP production and bioenergetic insufficiency.^32^ In skeletal muscle, oxidative phosphorylation (OXPHOS) is notably impaired, characterized by reduced activity of mitochondrial respiratory complexes I and II, and a diminished maximal OXPHOS capacity strongly linked to frailty.^33^ This cellular energy crisis directly precipitates muscle atrophy and weakness.^34^^,^^35^ Translational research further underscores the metabolic dimension of this dysfunction. In animal models, a decline in alpha-ketoglutarate (α-KG), a key TCA cycle intermediate, has been identified as a unique predictor of future frailty. The depletion of this metabolite may both reflect and contribute to the observed mitochondrial and TCA cycle abnormalities in human frailty, highlighting a promising target for mechanistic investigation.^36^

### Neurological dysfunction and cognitive decline

Neurological dysfunction discussed here is defined as a potential mechanistic contributor to frailty-related vulnerability, rather than an independent defining feature of frailty phenotype. Neuropathological changes such as hippocampal neuronal loss, synaptic dysfunction, and accumulation of hyperphosphorylated tau and amyloid-β are well-documented contributors to cognitive decline in aging and neurodegenerative diseases.^37^^,^^38^ Concurrently, excessive activation of microglia, the resident immune cells of the central nervous system, leads to sustained release of pro-inflammatory cytokines (e.g., IL-1β and TNF-α), which further exacerbates neuronal damage and impairs synaptic plasticity.^39^^,^^40^ These mechanisms are particularly prominent in Alzheimer’s disease and MCI, where neuroinflammation and neurodegeneration co-drive cognitive deterioration.^41^

Neuroimaging studies have provided structural evidence for these processes, reporting that frail individuals exhibit increased white matter hyperintensities and reduced gray matter volume, particularly in subcortical regions. These structural and functional brain changes are associated with vulnerability across both physical frailty and cognitive frailty domains, although further longitudinal studies are needed to clarify the directionality and underlying mechanisms of these relationships.^42^^,^^43^

### Metabolic dysregulation and endocrine signaling

In parallel with neurological alterations, metabolic dysregulation represents another possible pathway associated with frailty-related vulnerability. Epidemiological evidence suggests that MetS is associated with a higher likelihood of frailty. Longitudinal analysis further showed that each one-standard-deviation increase in cumulative MetS score corresponded to a 22.2% rise in frailty risk (OR = 1.222, 95% CI: 1.133–1.319).^14^

More specifically, frailty has been linked to disordered lipid and cholesterol metabolism. Yan et al.^36^ used the atherogenic index of plasma (AIP), a composite marker of atherogenic lipid profiles and systemic inflammation, and demonstrated that higher AIP independently predicts frailty, especially in women. In addition, emerging metabolomic studies in animal models reveal pronounced metabolic disturbances during frailty progression, particularly in tryptophan metabolism, with metabolites such as kynurenine and quinolinate elevated in frail females. Moreover, studies also highlight sex-specific metabolic patterns: Frail females show shifts in B-vitamin metabolism (e.g., flavin adenine dinucleotide and pyridoxate), whereas frail males exhibit stronger associations with alterations in sphingomyelins and glycerophosphoethanolamine metabolism.^44^ These findings support the view that metabolic dysregulation may contribute to frailty-related heterogeneity, while also underscoring the need to distinguish biological risk pathways from data-driven subphenotypes described elsewhere in this review.

Endocrine signaling may represent another important component of this metabolic network. In particular, reduced insulin-like growth factor-1 (IGF-(1) levels are independently associated with frailty, potentially through pathways related to muscle metabolism and protein turnover, including phosphoinositide 3-kinase (PI3K)/protein kinase B/mechanistic target of rapamycin pathway.^45^^,^^46^^,^^47^ However, some studies claim that they found no clear association between serum IGF-1 levels and appendicular skeletal muscle mass index or grip strength in older women,^48^ and there is evidence that other PI3K-mediated pathways may be involved in muscle strength decline, leaving the direct relationship between this pathway and muscle strength controversial.^49^^,^^50^ Nonetheless, IGF-1 is increasingly recognized as a central hub linking nutrition, inflammation, and frailty, reflecting protein synthesis and overall nutritional status, and also regulating autophagy and inflammation-related gene expression to delay frailty progression.^51^^,^^52^^,^^53^^,^^54^ However, further research is needed to clarify its dynamic role.

### Genetic and epigenetic regulatory networks

Frailty arises from a complex interplay of genetic and epigenetic factors that remain only partially delineated. A landmark meta-analysis of genome-wide association studies, integrating data from the UK Biobank (*n* = 164,610; ages 60–70) and the Swedish TwinGene cohort (*n* = 10,616; ages 41–87), identified 14 independent loci significantly associated with the FI. These loci are enriched in pathways critical for neurological integrity and metabolic regulation. The aggregate heritability of frailty attributable to common single-nucleotide polymorphisms was estimated at 11%. Subsequent transcriptomic enrichment analyses further revealed significant downregulation of gene sets predominantly expressed in the frontal cortex and hippocampus, implicating these brain regions in frailty pathogenesis.^49^ At the level of candidate genes, functional polymorphisms such as rs2794520 in the CRP gene have been associated with increased frailty risk, providing mechanistic support for a genetic link between inflammatory diathesis and frailty susceptibility.^50^

Moreover, frailty exhibits a strong molecular correlation with multiple established hallmarks of biological aging. Key associations include: reduced mitochondrial DNA copy number (indicative of genomic instability and bioenergetic decline); telomere attrition; altered global DNA methylation profiles (epigenetic drift); decreased abundance of molecular chaperones heat shock protein 70 (Hsp70) and heat shock protein 72 (Hsp72) (loss of proteostasis); diminished signaling of IGF-1 and sirtuin 1 (SIRT1) (dysregulated nutrient sensing); elevated circulating growth differentiation factor-15 (GDF-(15) (a pleiotropic marker of mitochondrial dysfunction, cellular senescence, and aberrant intercellular communication); imbalanced CD4+/CD8+ T cell ratios (immunosenescence); and reduced levels of circulating osteoprogenitor cells (stem cell exhaustion).^55^^,^^56^ In summary, these findings converge to conceptualize frailty not as a discrete disease entity, but as a systemic phenotype emerging from the progressive, interconnected failure of multiple genetic, epigenetic, and physiological systems that define the aging process itself.

### Other elements

In summary, the onset and progression of frailty syndrome result from multifactorial interactions. A large-scale cohort study (*n* = 483,033), which conducted a median follow-up of 9 years among participants aged 38–73, found that frailty severity was significantly associated with cognitive decline, increased early-life risk, unhealthy lifestyle, reduced physical fitness, increased psychological symptoms, severe environmental pollution, and adverse biochemical markers, some of which showed bidirectional associations with the frailty.^42^ This suggests that frailty not only relates to physiological decline but is also influenced by psychological status and social support systems. Physiologically, frailty is significantly associated with chronic inflammation, oxidative stress, malnutrition, and sarcopenia. In terms of psychological and social factors, cognitive decline, mood disorders, and social isolation may contribute to frailty. Unhealthy lifestyles are also likely to contribute to frailty. Sedentary behavior, insufficient physical activity, and sleep disorders—behaviors that fail to meet the 24-h movement guidelines have been associated with increased frailty risk.^57^

Therefore, early identification and intervention targeting these modifiable psychological and social risk factors are crucial for the prevention and delay of frailty.

These biological alterations mentioned above collectively contribute to functional decline and increased vulnerability in older adults. Despite extensive investigations into these influencing factors and mechanistic pathways, these factors and pathways do not operate in isolation. Instead, they form a complex interactive network. Future research efforts should gradually transition from a focus on individual factors to a more systematic, network-based approach. This involves leveraging systems biology methodologies to uncover the critical intersection points among inflammation, metabolism, and neural function. These intersection points may hold greater potential as intervention targets compared to individual pathways. A deeper understanding of these mechanisms provides the scientific basis for developing precise diagnostic tools that can capture frailty in a dynamic, multisystem way. Therefore, the following section focuses on emerging diagnostic approaches that translate biological insights into clinical assessment.

## Diagnostic approaches

### Clinical tools

Multiple clinical tools have been developed to assess frailty, each capturing distinct dimensions of the syndrome. Among phenotype-based models, the fried frailty phenotype is the most widely adopted. It operationalizes frailty using five physical criteria: unintentional weight loss, weakness (measured by grip strength), self-reported exhaustion, slow walking speed, and low physical activity. An individual meeting three or more criteria is classified as frail, while one or two criteria indicate a pre-frail state.^58^ This model is extensively validated and applied. It has been implemented in 164 clinical trials, functioning both as a screening instrument to identify target populations (90 studies) and as an outcome measure to evaluate intervention efficacy (81 studies).^59^ Its effectiveness in assessing physical frailty is further supported by large-scale epidemiological evidence, such as a study involving 189,898 women from the UK Biobank.^2^ Due to its simplicity and well-established predictive value for adverse health outcomes, the Fried Phenotype remains a cornerstone in both research and epidemiological contexts.

In contrast, deficit accumulation models, exemplified by the FI , conceptualize frailty as the proportion of health deficits an individual has accumulated over time.^60^^,^^61^ These deficits encompass multiple domains, including diseases, symptoms, functional impairments, and laboratory abnormalities, typically comprising 30 to 70 items, though the exact number varies across studies.^62^^,^^63^ This approach offers a more comprehensive assessment and has demonstrated superior accuracy in predicting mortality compared to phenotype-based tools.^64^ However, its requirement for more extensive data collection can limit feasibility in routine clinical or resource-constrained settings.

Other instruments, such as the clinical frailty scale (CFS), approach frailty from a different perspective. The CFS is a validated 9-point scale that assesses frailty based on clinical evaluation. It is particularly useful for rapid screening in emergency and perioperative contexts. Scores range from 1 (very fit) to 9 (terminally ill), with higher scores indicating greater frailty.^65^ A prospective multinational cohort study across 242 intensive care units (ICUs) in 22 countries, which enrolled 3,920 acutely hospitalized patients aged 80 years or older with 6-month follow-up, demonstrated that frailty (defined as CFS >(4) was a significant independent predictor of 30-day mortality, along with cognitive impairment (Informant Questionnaire on Cognitive Decline in the Elderly [IQCODE] ≥ 3.(5) and disability (Katz activities of daily living [ADL] ≤ 4).^66^

Each assessment tool presents distinct advantages and limitations (summarized in Table 1). The choice of instrument should therefore be tailored to the specific clinical context, research objectives, and available resources.

**Table 1:** Comparison of three commonly used frailty assessment models

Assessment Model	Theoretical Basis	Assessment Content	Strengths	Limitations	Suitable Scenarios
Phenotype (Fried Frailty Criteria)	frailty is an independent clinical syndrome characterized by physical decline and functional deterioration	five criteria: unintentional weight loss, weakness (grip strength), exhaustion, slow walking speed, and low physical activity	simple and easy to implement; strong predictive value for adverse outcomes	focuses only on physical aspects; neglects psychological and social factors; requires objective measurements	epidemiological studies, large-scale screening
Frailty Index (FI)	deficit accumulation theory: frailty results from the progressive accumulation of health deficits due to multi-system decline	30–70 health deficits (diseases, symptoms, functional impairments, laboratory abnormalities, and so forth)	comprehensive, multidimensional evaluation; strong predictive accuracy	requires extensive data collection; time-consuming	research settings, comprehensive assessments
Clinical Frailty Scale (CFS)	clinical global impression: frailty reflects overall health and functional status	9-point scale (from very fit to severely frail, including terminally ill)	rapid assessment; suitable for frontline clinical settings	subjective; requires experienced assessors	quick grading in clinical practice, emergency departments, and ICUs

### Biomarkers and laboratory test

Biomarker and laboratory-based assessments provide objective measures that complement the clinical evaluation of frailty (Table 2). While prior reviews have comprehensively summarized the evidence for core frailty-related biomarkers, particularly inflammatory cytokines, the present review aims to highlight a broader, multidimensional biological profile.^22^^,^^71^^,^^72^ In this context, biomarkers should be viewed not as stand-alone diagnostic substitutes, but as indicators that may offer mechanistic insight, support clinical assessment, and, in some cases, aid risk stratification.

**Table 2:** Summary of key biomarkers and laboratory indicators associated with frailty

Biomarker type	Representative markers	Primary role in frailty assessment	Pathophysiological/clinical relevance	Evidence summary	Key references
Inflammatory markers	IL-6, CRP, TNF-α	mechanistic insight; risk stratification	reflect chronic low-grade inflammation	consistently elevated in frailty; predictive of functional decline	El Assar et al.^22^
Oxidative stress markers	d-ROMs, BAP, MDA	mechanistic insight; supportive biomarker	indicate oxidative damage/antioxidant capacity	higher d-ROMs, lower BAP linked to frailty risk	Saum, Soysal et al.^67^^,^^68^
Nutritional/metabolic markers	albumin, prealbumin, Hb, 25OHD	clinical supportive marker	reflect nutrition, anemia, and vitamin D status	low levels associated with frailty onset and severity	Liang, Ju et al.^69^^,^^70^
Omics-based markers	altered amino acid profile, inflammatory proteome	early detection; risk stratification; mechanistic profiling	potential early detection, risk stratification	merging evidence from metabolomics/proteomics	Pan, Salvioli et al.^55^^,^^71^

IL-6, interleukin-6; CRP, C-reactive protein; TNF-α, tumor necrosis factor-alpha; d-ROMs, derivatives of reactive oxygen metabolites; BAP, biological antioxidant potential; MDA, malondialdehyde; Hb, hemoglobin; 25OHD, 25-hydroxyvitamin D.

Inflammatory markers remain the most consistently reported associations, with elevated levels of IL-6, CRP, and TNF-α being linked to an increased risk of frailty and adverse outcomes.^22^ These markers reflect a state of chronic low-grade inflammation, which is a central component of frailty pathophysiology.

Beyond inflammation, oxidative stress markers provide a complementary view

into frailty-related biological dysregulation. Indicators such as d-ROMs and biological antioxidant potential (BAP) have been proposed as sensitive predictors of physical decline in older adults.^67^^,^^68^ A disturbance in the oxidative balance is thought to contribute to muscle catabolism and impaired tissue repair.

Nutritional and metabolic markers, including serum albumin, prealbumin, vitamin D, and hemoglobin, remain clinically relevant because they reflect nutritional reserve, anemia, and metabolic vulnerability—factors that frequently coexist with frailty and may influence its severity.^69^^,^^70^ Furthermore, advances in omics technologies have facilitated the discovery of potential biomarkers, such as altered amino acid profiles and specific inflammatory proteins, which may capture frailty-related heterogeneity earlier than traditional laboratory measures alone. For instance, Pan et al.^71^ reviewed omics-based biomarker discovery for frailty, highlighting characteristic metabolic shifts and inflammatory signatures. Salvioli et al.^55^ emphasized the importance of multi-dimensional markers in the context of aging and frailty, encompassing inflammation, oxidative stress, muscle function, and microRNAs. Integrating biomarker profiles with established clinical assessment tools holds promise for enhancing diagnostic precision and informing the development of personalized intervention strategies.

### Imaging and functional tests

The complex pathophysiology of frailty and the absence of a universally accepted comprehensive definition pose challenges for its characterization using single or even composite biomarker panels. Imaging and functional assessments complement biomarker-based approaches by providing quantitative information on musculoskeletal decline, organ-specific vulnerability, and multisystem changes related to frailty.

#### Musculoskeletal imaging and functional assessment

Sarcopenia, a key component of physical frailty, is defined by the concurrent presence of low muscle mass and impaired muscle function, including reduced muscle strength and/or physical performance.^73^ The assessment of sarcopenia-related changes is central to the evaluation of frailty based on imaging. Characterized as practical and radiation-free, muscle ultrasound is employed to evaluate muscle quantity and quality. Studies have demonstrated a significant association between rectus femoris thickness and FI scores.^74^ Cross-sectional imaging, including computed tomography (CT) and magnetic resonance imaging (MRI), allows precise quantification of muscle cross-sectional area and is widely used in research settings, particularly in high-risk populations such as candidates for liver transplantation, where both frailty and sarcopenia prevalence is notably elevated and independently associated with adverse clinical outcomes.^75^ The cross-sectional area of core muscles, such as the psoas muscle at the L4 vertebral level, has been proposed as an objective indicator of frailty severity.^76^ However, CT and MRI are not universally regarded as gold standards for clinical sarcopenia assessment; whole-body approaches and composite functional integration are increasingly emphasized in contemporary guidelines. Dual-energy X-ray absorptiometry (DXA) and bioelectrical impedance analysis (BIA) offer more accessible alternatives for estimating appendicular muscle mass in both clinical and epidemiological contexts.^73^^,^^75^^,^^77^

Overall, muscle mass assessment should be complemented by functional evaluation. Grip strength, measured by dynamometry, is a widely validated, cost-effective indicator of overall muscle function and an independent predictor of adverse outcomes in older adults. Physical performance measures, including gait speed and the short physical performance battery (SPPB), also provide additional functional dimensions that primarily reflect lower extremity neuromuscular function, balance, and overall mobility capacity.^8^^,^^78^^,^^79^ Together, these functional tests are indispensable complements to imaging-based mass quantification in a comprehensive sarcopenia and frailty assessment.

#### Multi-organ imaging assessment

Beyond skeletal muscle, frailty is associated with subclinical structural and functional alterations across multiple organ systems. Speckle-tracking echocardiography has revealed subclinical myocardial dysfunction in community-dwelling older adults without overt cardiovascular disease. A worsening of left ventricular longitudinal strain is associated with frailty, reflecting a diminished cardiac functional reserve.^75^ These findings suggest that subclinical cardiac dysfunction may be associated with the reduced physiological reserve characteristic of frailty, and that echocardiographic parameters could serve as complementary indicators of cardiovascular frailty-related vulnerability. However, cardiac imaging discussed here should be interpreted as one example of organ-specific vulnerability rather than as a uniquely privileged imaging domain.

Alterations in brain structure are increasingly recognized as correlates of frailty. A reduction in white matter integrity across multiple brain regions has been statistically associated with frailty progression over time.^80^^,^^81^ However, other research indicates that a modified brain frailty score, incorporating features such as white matter hyperintensities, enlarged perivascular spaces, chronic vascular lesions, and cerebral microbleeds, did not predict surgical outcomes in patients with moyamoya disease.^82^

### Wearable and digital health tools

The rapid advancement of wearable sensors, mobile applications, and telemonitoring platforms has created new possibilities for the early detection and continuous monitoring of frailty. Modern wearable devices, including smartwatches, fitness bands, and sensor-embedded footwear, serve as digital tools that enable the objective quantification of gait speed, stride variability, and activity patterns. These parameters function as digital biomarkers, offering continuous, real-world data that complement traditional clinical assessments and may capture subtle functional decline before it becomes clinically apparent.^83^^,^^84^^,^^85^

Inertial measurement unit (IMU)-based wearable sensors have shown consistent utility in detecting gait alterations associated with frailty. A previous study showed that gait speed, stride and step timing, double-support time, and daily step-related activity have been the significant and consistently discriminative parameters for frailty diagnosis.^86^ Compared with robust older adults, (pre)frail individuals generally exhibit slower gait, shorter step length, lower cadence, and more fragmented walking patterns, both in structured assessments and in free-living environments. Importantly, wearable-derived daily physical activity metrics appear to add discriminative value beyond gait parameters alone, especially for identifying pre-frailty.^87^ These findings support wearable-derived gait and activity metrics as objective and ecologically valid complements to conventional frailty assessment, with promise for the early detection of pre-frailty.

App-based and telemonitoring platforms extend these capabilities by integrating multiple data streams into structured frailty assessment frameworks. The e-health application “CARE,” for example, incorporates a frailty module for status assessment and an activity module for fall risk evaluation. In a study of 1,151 older adults from the Quebec Longitudinal Study on Nutrition and Successful Aging, validated critical thresholds for the CARE FI were established: scores ≥0.24 indicate frailty, scores <0.05 indicate robustness, and scores between 0.06 and 0.23 classify pre-frailty. Both frailty status identified by the CARE frailty module and high fall risk identified by its activity module were significantly associated with actual fall outcomes, with a notable association observed between CARE-identified high fall risk and severe falls in frail individuals.^88^

Collectively, wearable and digital health tools offer complementary approaches to frailty assessment. Their potential value lies in providing objective, real-world, and potentially longitudinal data that may augment conventional clinical evaluation. Future work may need to focus on improving the integration and interpretation of wearable-derived data, while current evidence remains heterogeneous and requires stronger validation.

### Toward quantifying systemic resilience: A complementary framework

The diagnostic approaches discussed above primarily characterize frailty as a static state of vulnerability. However, a more clinically valuable paradigm lies in quantifying systemic resilience, defined as the capacity of an organism to maintain or rapidly recover function when challenged by a physiological or psychological stressor.^89^ This distinction has profound clinical implications, as quantifying resilience may enable earlier risk identification and real-time monitoring of intervention effectiveness.

Quantifying resilience requires moving beyond static, one-time assessments toward dynamic stress tests that measure the body’s response to and recovery from standardized challenges. Emerging metrics in this domain include:

Physiological recovery time. The capacity of the cardiovascular and autonomic nervous systems to recover from physiological challenge represents a clinically meaningful indicator of systemic resilience. Research found that frail older adults exhibit significantly impaired autonomic function, including blunted orthostatic heart rate responses and reduced heart rate variability, reflecting a diminished capacity to mount and sustain adaptive responses to physiological stress.^90^ Consequently, the time course of cardiovascular recovery following standardized physical or cognitive challenges serves as a pragmatic, quantifiable metric, where prolonged recovery kinetics identify individuals at heightened risk of functional decline.

Dynamic cardiovascular challenges. Orthostatic challenges, such as heart rate and blood pressure responses to standing, assess autonomic and cardiovascular resilience. The coexistence of orthostatic hypotension and frailty synergistically increases mortality hazard. As frailty is strongly linked to an increased risk of impaired orthostatic stabilization, such testing functions as both a sensitive marker of diminished cardiovascular resilience and a prognostic tool for adverse outcomes in aging populations.^91^^,^^92^

Motor-cognitive stress tests. Dual-task gait tests, which assess walking performance under cognitive load, can quantify the reserve capacity of the motor-cognitive system. Frail individuals typically exhibit significantly lower gait speeds and higher dual-task costs under cognitive load compared to robust peers.^93^^,^^94^ The degree of performance decrement under dual-task conditions, and the speed of recovery afterward, may reflect resilience in the integrated neural control of movement.

Composite resilience scores. Integrating data from multiple domains, such as inflammatory markers (e.g., CRP and IL-6), mitochondrial function (via respirometry or surrogate markers such as GDF-15), and digital biomarkers of physical activity, may yield a multidimensional resilience index that outperforms any single measure. For example, the rate at which step counts return to baseline after an acute illness or hospitalization, passively monitored via wearables, could serve as a real-world, ecologically valid measure of resilience.^22^^,^^55^^,^^84^

Digital health technologies, as further discussed in Section 7.2, offer promising opportunities to operationalize these resilience metrics in free-living settings. By embedding resilience quantification into clinical practice, we can expand beyond a reactive model of managing established frailty to also encompass a proactive model of monitoring and enhancing recovery capacity. This complementary framework, integrated throughout this review, directly informs the preventive and therapeutic strategies discussed in the following section.

## Preventive and therapeutic strategies

**Figure 2:**
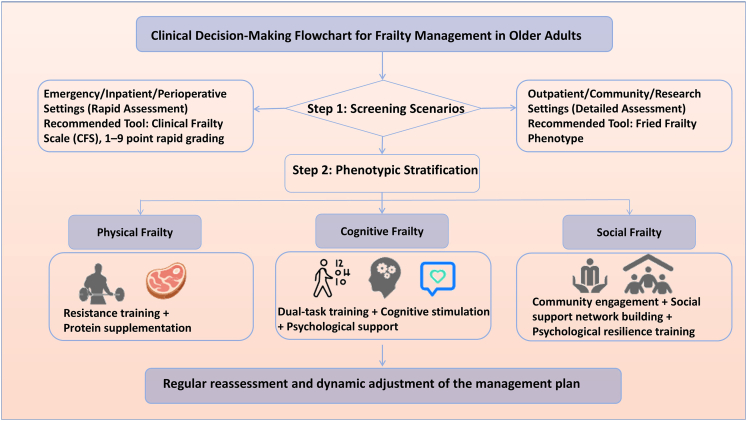
Clinical decision-making flowchart for frailty management in older Adults. This flowchart summarizes a structured approach to frailty management, including screening, multidimensional assessment, frailty stratification, and individualized multidomain interventions. Core strategies include exercise, nutrition, cognitive-psychological support, medication optimization, and multidisciplinary care.

Building upon evidence from diagnostic and mechanistic research, interventions for frailty have progressively shifted toward multidomain and personalized frameworks. Effective frailty-oriented care and tailored clinical management necessitate the integration of age-related health outcomes, individual risk factors, patient-centered goals, and evidence-based guidelines. As outlined by the New England Journal of Medicine (NEJM),^2^ the primary objectives of management are to augment physiological reserve, enhance resilience and recovery capacity, and to prevent or attenuate the impact of stressors. A tiered approach to intervention is recommended, with specific measures corresponding to the stages outlined in Table 3. Based on the screening tools and phenotypic stratification discussed above, the main objective of clinical management is the implementation of targeted intervention. To facilitate the translation of these conceptual frameworks into clinical practice, we propose a comprehensive decision-making pathway. As conceptually summarized in Figure 2, this pathway guides clinicians through setting-specific screening, phenotypic stratification, and the implementation of targeted, multidomain interventions.

**Table 3:** Frailty stratification and corresponding management strategies

		Healthy	Pre-frail	Frail	Advanced Frail
Frailty Score t	fried frailty phenotype	0	1-2	3-4	5
	frailty index (FI)	<0.08	0.08-0.24	0.25-0.45	≥0.45
	Clinical Frailty Scale (CFS)	1-3	4	5-7	8-9
Goal		optimize physiological reserve; prevent frailty onset	optimize physiological reserve; reverse pre-frailty	enhance resilience and recovery capacity; slow functional decline	prioritize comfort and quality of life
Lifestyle		physical exercise and training high-quality diet active social engagement	appropriate physical activity enhanced protein intake active social engagement	moderate-intensity exercise that is well tolerated high-quality diet (protein intake) maintain social contact as feasible	tolerable physical activity flexible nutrition strategy social engagement as appropriate
Disease Management		disease-specific guidelines	disease-specific guidelines	balance treatment benefit and burden	de-escalate therapy as appropriate
Preventive Care		vaccinations; routine cancer screening; fall prevention counseling; bone health assessment	vaccinations; routine cancer screening; fall risk assessment; osteoporosis screening; nutritional screening	vaccinations; individualized cancer screening based on life expectancy, burden, and likely benefit; fall prevention; pressure ulcer prevention; medication review	vaccinations; selective discontinuation of low-value screening; comfort-focused care; advance care planning
Intervention Measures		______	address reversible factors exercise nutrition support CGA medication review	address reversible factorsRehabilitation (PT/OT) nutrition support CGA and multidisciplinary intervention medication review	medication review

FI cutoffs are approximate and may vary across instruments; ≥0.45 is considered to approach the theoretical upper limit.^95^^,^^96^

### Exercise therapy

Exercise intervention is among the most well-supported and feasible strategies for reducing frailty-related vulnerability and improving physical function in older adults. A stepped-wedge cluster randomized trial conducted in China demonstrated that structured exercise programs were associated with both immediate and sustained improvements in frailty status, physical function, and psychosocial well-being among community-dwelling older adults.^97^ In parallel,a large-scale U.S. study (*n* = 5,726; mean age 50.25 ± 0.31 years) found that adherence to 24-h movement guidelines was significantly and inversely associated with frailty risk.^57^

Exercise may exert beneficial effects through multiple physiological pathways, which include the preservation of muscle mass and function, modulation of inflammatory and oxidative stress responses, enhancement of mitochondrial function and autophagy, and influences on neuroplasticity and insulin-related signaling. Targeted exercise modalities, including resistance, aerobic, balance, and flexibility training, each address distinct components of physical function.^98^ Accordingly, staged and progressive multicomponent exercise regimens are considered a core strategy for frailty prevention and management. These programs typically integrate resistance, strength, aerobic, flexibility, and balance training. The efficacy of such multimodal approaches is supported by the landmark SPRINTT randomized controlled trial, which demonstrated that a long-term multicomponent intervention integrating physical activity with technological support reduced the incidence of mobility disability in older adults with physical frailty and sarcopenia.^99^ Generally, multidimensional interventions lasting at least 12 weeks have been associated with improvement in frailty-related outcomes, with specific training modalities detailed in Table 4.

**Table 4:** Types of physical training and recommendations for frailty management

Type of Training	Core Element	Key Implementation	Recommended Protocol
Resistance Training	equipment-based, bodyweight,	progressive overload	2–3 sessions/week; 1–3 sets of 8–12 repetitions at moderate-to-high intensity (60–80% 1RM); allow ≥48h recovery between sessions
Aerobic Exercise	walking, jogging,	moderate, continuous activity	≥150 min/week; sustained over ≥3 months
Balance Training	single-leg stands, tandem walking,	safety and fall prevention	10-15min/day
Flexibility Training	dynamic and static stretching	incorporate post-exercise	5-10min/session

Recommended protocols represent targets for each training modality individually and are not intended to be performed cumulatively. For frail older adults, total weekly exercise should be individualized; priority should be given to resistance and balance training. Clinicians are advised to initiate at lower intensities and volumes, progressing gradually based on tolerance.

For sedentary or older individuals, initiation with single-component training is recommended, progressively advancing to more complex and multidimensional exercise routines. Both home-based and group-based exercise programs have been associated with a lower likelihood of frailty progression. Evidence suggests that high-intensity exercise yields superior outcomes in slowing the trajectory of frailty compared to low- or moderate-intensity physical activity.^100^ Prior to intervention, personalized endurance assessments, such as cardiopulmonary exercise testing or the 6-min walk test, may help inform individualized exercise prescriptions. Notably, exercise interventions sustained beyond three months are associated with more durable benefits.^101^

Emerging integrative approaches, such as combining aerobic exercise with cognitive training via virtual reality (VR), may further enhance functional status. Integrated VR-based rehabilitation, when implemented alongside structured exercise regimens, has shown promising results in frail older adult populations.^1^

### Cognitive and psychological interventions

Cognitive and psychological interventions are integral to comprehensive frailty management, particularly in addressing cognitive frailty—a condition increasingly recognized as a potential target for intervention, even in the context of neurodegenerative disorders such as Alzheimer’s disease.^12^ As Panza and colleagues have highlighted, targeting cognitive frailty may offer a strategic opportunity for the secondary prevention of dementia, given its position as a high-risk state for cognitive decline.^11^^,^^12^ These interventions encompass several key strategies: exercise-cognitive dual-task training, multimodal non-pharmacological approaches (incorporating nutritional support, psychological resilience enhancement, and social support), digital health tools, and targeted psychological therapies.

Dual-task training, which combines physical and cognitive demands, has shown promising results in improving cognitive function, psychological well-being, and quality of life in older adults with cognitive frailty. A retrospective study of 130 patients with coexisting cognitive frailty and depression found that an intervention group receiving dual-task training showed significant improvements in both cognitive performance and physical activity capacity.^102^ Similarly, VR-integrated motor-cognitive training has been associated with improvements in cognitive and physical function in older adults, with its benefits corroborated by multicenter randomized controlled trials.^103^

Multimodal non-pharmacological interventions represent another key focus. Psychological resilience, which buffers the negative impact of social frailty, is associated with a significantly reduced risk of cognitive decline in resilient individuals.^104^ Social support further contributes to this protective effect by indirectly lowering the risk of cognitive frailty through resilience enhancement.^105^

Digital health interventions are emerging as a promising avenue for managing cognitive frailty. Systematic reviews and meta-analyses indicate that such interventions are associated with significant improvements in global cognitive and executive function, while also reducing both frailty severity and depressive symptoms.^106^

Targeted psychological interventions focus on ameliorating emotional symptoms, fatigue, and social cognition. Cognitive behavioral therapy, for example, may be effective in alleviating depression and anxiety, mitigating fear of falling, and addressing other psychological issues, making it a suitable approach for frail older adults.^107^

Despite these advances, several challenges and future research directions persist. While early-stage cognitive frailty may be partially reversible, there remains an urgent need to develop more precise assessment tools, such as blood-based biomarkers, to facilitate earlier detection and enable more personalized and timely interventions.^107^^,^^108^

### Nutritional interventions

Adequate nutritional support is fundamental to counteracting the physiological decline associated with frailty. Evidence indicates that a high protein intake (1.0–1.5 g/kg/day), when combined with vitamin D supplementation, may help attenuate muscle catabolism. Protein supplementation addresses frailty-related deficiencies in specific amino acids, such as valine, leucine, isoleucine, and lysine. This can be achieved through dietary means, including increased consumption of low-fat milk or yogurt.

For frail and multimorbid older adults with deficiencies in vitamins and caloric nutrients, targeted supplementation of vitamins and other essential micronutrients is recommended. Additionally, maintaining good oral hygiene to preserve chewing function is an important yet often overlooked aspect of nutritional management in this population.^101^

### Pharmacological and comprehensive management

A cornerstone of comprehensive frailty management involves the judicious review and optimization of medication regimens. Reducing polypharmacy and discontinuing potentially inappropriate medications, which is guided by validated tools such as the Screening Tool of Older Persons’ Prescriptions/Screening Tool to Alert to Right Treatment (STOPP/START) criteria, can significantly mitigate adverse outcomes such as delirium and falls.^109^

While no pharmacological agent is currently FDA-approved specifically for frailty, the research landscape is shifting from single-target hormonal therapies to broad-spectrum metabolic modulators. Historically, hormonal interventions, including selective estrogen receptor modulators such as raloxifene and tibolone, have yielded mixed results. Although early trials indicated that agents such as raloxifene could improve bone mineral density, they have failed to consistently translate into enhanced muscle strength or physical performance in older women, while carrying potential risks of thromboembolic or cerebrovascular events. Because frailty is fundamentally driven by multisystem dysregulation instead of single musculoskeletal deficits, traditional single-target interventions have often failed to translate increased tissue mass into enhanced systemic resilience.^110^ Therefore, current research increasingly focuses on repurposing metabolic drugs, particularly with the higher global focus on agents that modulate cardiometabolic pathways.

SGLT2 inhibitors: Originally developed for diabetes, sodium-glucose cotransporter-2 (SGLT2) inhibitors have demonstrated pleiotropic effects beyond glycemic control, including improved mitochondrial function, reduced inflammation, enhanced autophagy, and favorable shifts in energy metabolism.^111^^,^^112^ These mechanisms directly target several key pathophysiological pathways of frailty discussed in Section 4 (e.g., mitochondrial dysfunction and chronic inflammation). Clinical studies in patients with heart failure and diabetes have shown that SGLT2 inhibitors improve physical function, reduce hospitalizations, and slow frailty progression.^113^ Their potential to enhance systemic resilience—by improving cardiac and renal function while mitigating metabolic stress—makes them a promising candidate for frailty management, although dedicated trials in frail, non-diabetic older adults are urgently needed.

GLP-1 receptor agonists: Glucagon-like peptide-1 (GLP-(1) receptor agonists are under intense investigation for their pleiotropic effects on weight loss, inflammation, and insulin sensitivity.^114^^,^^115^^,^^116^ By reducing visceral adiposity and systemic inflammation, these agents may indirectly benefit physical function and metabolic health in frail individuals. However, their impact on muscle mass is a double-edged sword: While they effectively reduce fat mass, rapid weight loss can accelerate sarcopenia if not accompanied by adequate protein intake and resistance exercise.^117^ Therefore, in frail older adults, GLP-1 receptor agonists should be used with caution, with close monitoring of muscle mass and physical function, and ideally combined with structured exercise and nutritional support.

Ultimately, the future of pharmacological management lies not in single-target agents, but in developing personalized strategies that target the fundamental biological drivers of frailty. The emerging evidence for SGLT2 inhibitors and GLP-1 receptor agonists illustrates the potential of repurposing metabolic drugs, but also underscores the need for rigorous, frailty-specific trials that assess not only efficacy but also safety and impact on muscle health.

### Multidisciplinary team (MDT) interventions

The Comprehensive Geriatric Assessment (CGA) is a foundational MDT-driven approach that systematically integrates medical, pharmacological, rehabilitation, nutritional, and psychosocial support to formulate individualized care plans for frail older adults, ultimately aiming to improve their quality of life.^110^ Its value is evident in specific high-risk clinical scenarios. For example, among frail patients undergoing cardiac surgery, the implementation of preoperative MDT care has been associated with meaningful adjustments in perioperative management and a concomitant reduction in the risk of major complications.^118^

While the combined “exercise and nutrition” paradigm remains the cornerstone of frailty management, its benefits demonstrate considerable heterogeneity across individuals. This underscores a critical avenue for future research, which may need to increasingly focus on addressing a more precise question: Which individual (frailty subtype), at what disease stage or time point, will derive the greatest benefit from which specific combination of interventions? Answering this necessitates a shift in clinical trial design. Future randomized controlled trials should be stratified by frailty subtypes and other relevant phenotypes. Consequently, the focus of public health efforts should evolve from universal community screening toward targeted preventive interventions for identified high-risk subgroups, such as patients with metabolic syndrome.

## Future directions and research outlook

Although significant progress has been made in elucidating the biological underpinnings and clinical management of frailty, the field remains somewhat descriptive and fragmented. Traditional phenotype-based tools remain highly valuable because they are practical, validated, and feasible for routine clinical use, broad screening, and many resource-limited or acute care settings.^2^ However, these approaches have inherent limitations, as they are primarily optimized for cross-sectional classification and may be less sensitive to subclinical, multisystem changes before apparent functional decline appears. Therefore, future research may need to further extend traditional phenotype-based frameworks toward more dynamic, mechanism-driven, and technology-enabled models in order to complement traditional assessments. Complex multi-omics or digital profiling models are more justifiable in research settings or when tailoring interventions for specific high-risk subgroups, where the incremental clinical benefit is likely to outweigh the added complexity.^2^^,^^57^ Importantly, it must be acknowledged that direct comparative evidence demonstrating the superiority of complex multi-omics or digital profiling models over traditional tools in real-world clinical outcomes remains limited, and rigorous validation studies are urgently needed.

### Systems biology and multi-omics integration

Current mechanistic research has often focused on isolated biomarkers or single organ systems. However, frailty reflects the cumulative dysregulation of multiple biological networks, including those involved in inflammation, mitochondrial metabolism, endocrine signaling, and neurodegeneration. Integrative multi-omics approaches that combine genomics, transcriptomics, metabolomics, and proteomics may offer a complementary avenue to identify biologically relevant pathways and molecular signatures associated with frailty trajectories. Longitudinal multi-omics studies, particularly when integrated with machine learning analytics, may further help clarify the crosstalk among immune, metabolic, and neurocognitive systems, and could support the development of novel, biologically interpretable frailty indices.^71^

### Precision phenotyping and digital health integration

Traditional frailty assessments, such as the fried frailty phenotype or the FI, capture limited aspects of vulnerability. Advances in wearable sensors, gait analysis, and continuous physiological monitoring may support dynamic frailty phenotyping. The integration of digital biomarkers with clinical and biological data may provide additional opportunities for real-time risk assessment and individualized intervention. Algorithms based on artificial intelligence can analyze multidimensional datasets to derive predictive digital models of frailty that simulate disease trajectories and guide personalized therapy.^88^^,^^119^ The potential for complex profiling to support earlier detection rests on several complementary mechanisms. Molecular alterations, such as shifts in metabolomic profiles or inflammatory proteomes, may emerge before clinically detectable functional decline, thereby offering a biochemical window that phenotype-based criteria may not capture.^71^ Wearable-derived gait and activity metrics, by providing continuous free-living data, can detect subtle deterioration invisible to clinic-based snapshot assessments.^84^ Together, these approaches may shift the detection window earlier in the pathophysiological timeline, enabling intervention before the syndrome becomes clinically manifest.

### Mechanism-driven and personalized interventions

Multimodal interventions incorporating exercise, nutrition, and psychological components remain the cornerstone of frailty management, yet most clinical trials adopt a standardized approach. Future studies may benefit from adaptive trial designs that link biological endophenotypes to specific interventions. For example, mitochondrial dysfunction could be targeted through exercise-induced modulation of myokines, or chronic inflammation could be addressed via tailored nutritional or pharmacological anti-inflammatory strategies.^97^ Integrating psychosocial and behavioral determinants within these biological frameworks will support the development of truly personalized care plans.

### Translational and policy implications

To bridge the gap between research and practice, frailty management should be progressively integrated into healthcare systems as a routine screening and decision-making tool. Developing scalable, low-cost screening platforms which integrate clinical, biomarker, and digital assessments may support earlier detection at the community level. From a resource planning perspective, evidence indicates that both the presence and progression of frailty are associated with increased utilization and higher costs of imaging services,^120^ underscoring the economic imperative for earlier, less resource-intensive identification strategies. Implementation should be tailored to resource availability and clinical purpose. Cross-sector collaboration among clinicians, data scientists, and policymakers will be essential for establishing precision public health models that address frailty as a continuum of systemic resilience loss.

### Frailty and resilience: Toward a more refined framework of healthy aging

Finally, frailty research may be expanded from describing vulnerability to understanding resilience, defined as the capacity to recover from physiological or psychological stress.^89^ Based on this evolving framework, frailty and resilience operate as related but distinct theoretical constructs. Frailty primarily characterizes a state of reduced physiological reserve and heightened susceptibility to adverse outcomes, while resilience reflects the dynamic capacity to respond to and recover from physiological or psychological stressors. Critically, these two dimensions are not mirror opposites: An individual may be frail by conventional criteria yet demonstrate meaningful recovery capacity following a specific acute challenge, while another may be classified as non-frail yet exhibit poor resilience when stressed. Thus, combining these interacting dimensions may form a more complete framework for characterizing vulnerability and recovery in older adults, which represents an important frontier for advancing geroscience and promoting healthy longevity.

## Conclusion

In summary, this review synthesizes evidence to reconceptualize frailty not as a static endpoint but as a dynamic and potentially modifiable process of declining systemic physiological reserve. The accumulated findings support an expanded framework advocated throughout this article: moving from the reactive management of frailty as a clinical syndrome toward its proactive prevention by enhancing resilience. To realize the future research directions outlined, particularly the development of predictive models informed by biological mechanisms and the implementation of prescriptive, multidomain interventions, sustained and interdisciplinary collaboration will be essential. By pursuing this roadmap, the field can move beyond descriptive frameworks and ultimately help transform the trajectory of aging for future generations.

## Acknowledgments

This work has been supported by the 10.13039/501100001809National Natural Science Foundation of China (no. 82471954 to Chunhong Hu), the Jiangsu Province Youth Science and Technology Talent Lifted Project (TJ-2023-028 to Yunyan Su), the Suzhou Medical Youth Backbone Talents-Mentor Pairing Project (Qngg2024001 to Yun Yan Su), and the Jiangsu Provincial Key Medical Discipline (JSDW202242 to Chun Hong Hu).

## Author contributions

Y.S., Y.G., and Y.H. contributed to the study concept and design, interpreted the data, and wrote and revised the manuscript.; R.C. contributed to interpreting the data and critical revision of the manuscript.; Y.Y. contributed to interpreting the data and critical revision of the manuscript.; C.H. contributed to the study concept and design, interpreting the data, and critical revision of the manuscript.; H.Y. contributed to the study concept and design, interpreted the data, and provided critical revision of the manuscript.; All authors reviewed and approved the final version, and no other person made a substantial contribution to the paper.

## Declaration of interests

The authors declare no competing interests.
